# Enrichment of HIV-1 Subtype AD Recombinants in a Ugandan Cohort of Severely Septic Patients

**DOI:** 10.1371/journal.pone.0048356

**Published:** 2012-10-29

**Authors:** Najah I. Doka, Shevin T. Jacob, Patrick Banura, Christopher C. Moore, David Meya, Harriet Mayanja-Kizza, Steven J. Reynolds, W. Michael Scheld, Wen Yuan

**Affiliations:** 1 Division of Infectious Diseases and International Health, Department of Medicine, University of Virginia, Charlottesville, Virginia, United States of America; 2 Myles H. Thaler Center for AIDS & Human Retroviruses Research, University of Virginia, Charlottesville, Virginia, United States of America; 3 Division of Allergy and Infectious Diseases, Department of Medicine, University of Washington, Seattle, Washington, United States of America; 4 International Respiratory and Severe Illness Center (INTERSECT), Department of Medicine, University of Washington, Seattle, Washington, United States of America; 5 Department of Community Health, Masaka Regional Referral Hospital, Masaka, Uganda; 6 Department of Medicine, Infectious Diseases Institute, Makerere University, Kampala, Uganda; 7 National Institute of Allergy and Infectious Diseases, National Institutes of Health, Bethesda, Maryland, United States of America; 8 Johns Hopkins School of Medicine, Baltimore, Maryland, United States of America; British Columbia Centre for Excellence in HIV/AIDS, Canada

## Abstract

**Background:**

Several population-wide HIV-1 subtype distribution studies in Uganda have evaluated relatively healthy clinic patients. Given the differences in HIV-1 disease progression based on subtype, we examined HIV-1 subtype distribution and disease outcomes among hospitalized patients with severe sepsis.

**Methods:**

Patients with severe sepsis were enrolled at two hospitals in Uganda. Data collected included demographics, Karnofsky scores, highly active antiretroviral therapy (HAART) use, HIV-1 serostatus, CD4+ T cell concentration, whole blood lactate concentration, and blood cultures. HIV-1 subtypes were determined by sequencing parts of the *gag* and *env* genes, followed by phylogenetic analysis.

**Results:**

Of the 267 patients evaluated, 228 (85.4%) were HIV infected. The predominant HIV-1 subtypes were A (46%), D (17%), and AD recombinants (30%). HIV-1 subtypes B, C, and other recombinants were uncommon. Patients infected with HIV-1 subtypes A, D and AD viruses were similar in demographics, CD4^+^ T cell concentration, HAART use, Karnofsky scores, whole blood lactate concentration, and positive blood cultures. There was no difference in 30-day mortality from severe sepsis between the 3 groups (p = 0.99).

**Conclusion:**

A high proportion of HIV-1 subtypes A and AD recombinants was observed in this cohort of severely septic patients. The proportion of AD recombinants was higher in this cohort than in previous cohorts of Ugandan HIV-1 patients. No difference in baseline demographics, clinical factors or 30-day mortality was seen across HIV-subtypes.

## Introduction

The Human Immunodeficiency Virus type 1 (HIV-1) pandemic is not only diverse in its genetic distribution [1], but also in pathogenesis, transmission and disease progression. HIV-1 is divided into 3 groups: Major (M), Outlier (O) and New (N). Group M is further subdivided into 9 major subtypes with varying global distribution [2]. In East Africa, the main subtypes encountered are A, D, and AD recombinants [3]–[8], with very few subtypes B, C or other recombinants [9], [10].

In Uganda, most studies investigating HIV-1 subtype distribution have evaluated relatively healthy patients seen in HIV clinics. To date, there are at least 9 studies that describe HIV-1 subtype distribution in Uganda between 1997 and 2009 with patient enrollment spanning 1989 through 2008 [3]–[12]. Subtypes A, D, and AD recombinants were predominantly identified. In the late 1980s to early 1990s, subtype D accounted for more than 50% of all subtypes, followed by subtype A [4], [5], [9]. In the mid 1990s to 2000s, there was an equal proportion of subtype A and D (∼47% each) [6], [7]. Recently, however, a higher proportion of subtype A compared to subtype D has been reported [8], [12].

The rate of disease progression among HIV-1 infected patients varies depending on subtype. Subtype D is associated with a faster rate of CD4^+^ T-cell decline and more rapid disease progression to AIDS and death compared to subtype A in both adult and pediatric HIV-infected populations in Uganda [13]–[15], Kenya [16] and Tanzania [17]. However, HIV-1 subtype does not always correlate with severity of HIV-associated illness. For instance, HIV-1 subtype association with neurocognitive performance has yielded conflicting results. In a Ugandan adult population, subtype D infection was associated with a higher prevalence of dementia compared to subtype A infection [18], while subtype A-infected Ugandan children demonstrated poorer neurocognitive performance compared to subtype D-infected children [19].

Sepsis from severe blood stream infections has been recognized as a significant cause of morbidity and mortality among patients infected with HIV-1. There is a 76–84% prevalence of HIV among patients who present with febrile illness or sepsis in sub-Saharan Africa [20]–[25], where the HIV pandemic has claimed most of its victims [26]. Given the association between certain HIV-1 subtypes and rate of CD4 decline and mortality, we sought to determine HIV-1 subtype distribution and mortality in a cohort of predominantly HIV-infected patients with severe sepsis in Uganda.

## Methods

### Study Design and PATIENT Recruitment

This was a prospective cohort study done in Uganda at Mulago Hospital, the national referral hospital in Kampala, and Masaka Regional Referral Hospital, 80 miles southwest of Kampala, Uganda. Between May 2008 and May 2009, we enrolled 426 severely septic patients admitted to the medical wards of these 2 hospitals in an intervention study of fluid resuscitation [27]. Adult patients (age ≥18 years) triaged to the hospitals’ Medical Casualty Units were enrolled based on the following inclusion criteria: (1) suspected infection as determined by the admitting medical officer; (2) two of the following: a) axillary temperature >37.5°C or <35.5°C, b) heart rate >90 beats/min, c) respiratory rate >20 breaths/min; (3) systolic blood pressure (SBP) ≤100 mmHg; and (4) either whole blood lactate concentration >2.5 mmol/L or Karnofsky Performance Scale (KPS) score ≤40. Patients with acute cerebrovascular events, gastrointestinal hemorrhage, suspected surgical or obstetric emergencies or shock without suspected infection were excluded.

**Figure 1:**
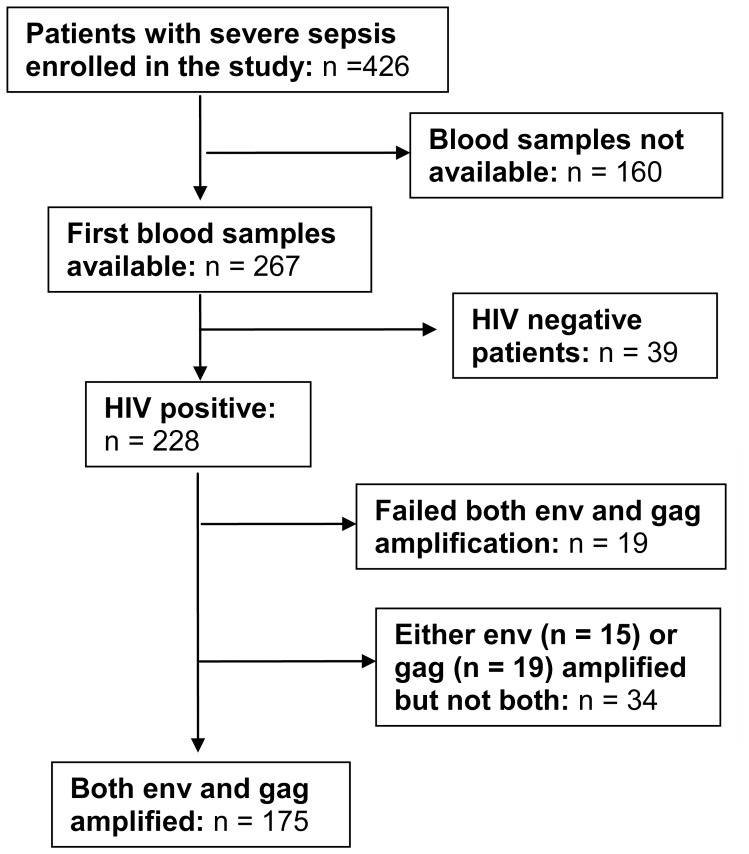
Enrollment procedure.

### Ethics Statement

Ethical approval was obtained from the research and/or ethics committees of the University of Virginia, Makerere University, Mulago Hospital, Infectious Diseases Institute, and Uganda National Council of Science and Technology. Written informed consent was obtained from each patient or a surrogate if the patient was too obtunded to provide consent.

### Data Collection and Clinical Assessments

At the time of enrollment, baseline information including demographic data (age, gender), clinical history (highly active antiretroviral therapy [HAART] use) and clinical data (body temperature, respiratory rate, blood pressure and KPS scores) were collected. Laboratory evaluation included: HIV serostatus, CD4+ T cell concentration (if HIV-infected), whole blood lactate concentration, and blood cultures. Thirty day mortality and length of hospital stay were also recorded.

### Viral RNA Extraction and Nested PCR Amplification of the *gag* and *env* Genes

Plasma samples were collected at admission, frozen and stored at −70°C, and shipped to the University of Virginia where viral RNA extraction, reverse transcription, and nested PCR amplification were performed. Viral RNA was extracted from 140 ul of plasma using the QiAmp Viral RNA mini kit (Qiagen) following the manufacturer’s instructions. Plasma obtained from patients receiving HAART was concentrated from 1 ml by ultracentrifugation at 30,000 x g for 1 hour at 4°C prior to RNA extraction. The extracted viral RNA was reverse transcribed into cDNA by the Superscript III reverse transcriptase (Invitrogen) following the manufacturer’s protocol. The primers used for the reverse transcription were 5′-TCCCTAAAAAATTAGCCTGYCT-3′ (2095-2074) and 5′-CCAATCAGGGAARWAGCCTTGTGTGT-3′ (9168-9143). The *env* gene C2–C4 region and the *gag* gene p24 region were amplified by nested PCR. The nested PCR primers for the *env* region amplification were 5′-CCAATTCCYATACATTATTG-3′ (outer sense primer, 6858–6877), 5′-TTRTATARTTCACTTCTCCA-3′ (outer antisense primer, 7678-7659), 5′-AGCACAGTACARTGYACACATGG-3′ (inner sense primer, 6951–6973), and 5′-ATRGGAGGGGCATAYATTGC-3′ (inner antisense primer, 7540-7521). The nested PCR primers for the *gag* region amplification were 5′- AGYCAAAATTAYCCYATAGT -3′(outer sense primer, 1174–1193), 5′- TCCTTYCCACATTTCCARCAGCC-3′ (outer antisense primer, 2045-2023), 5′- TGGGTRAAAGTAATAGARGA-3′ (inner sense primer, 1252–1271), and 5′- ACTCCCTGRCATGCTGTCATCATTTC-3′ (inner antisense primer,1847-1822). The numbers in parenthesis represent nucleotide positions in HIV HXB2 sequence. The nested PCR was run 35 cycles of 95°C for 30 sec, 50°C for 30 sec, and 72°C for 1 min. Amplified fragments were sequenced using an automated DNA sequencer at Genewiz Inc.

**Figure 2:**
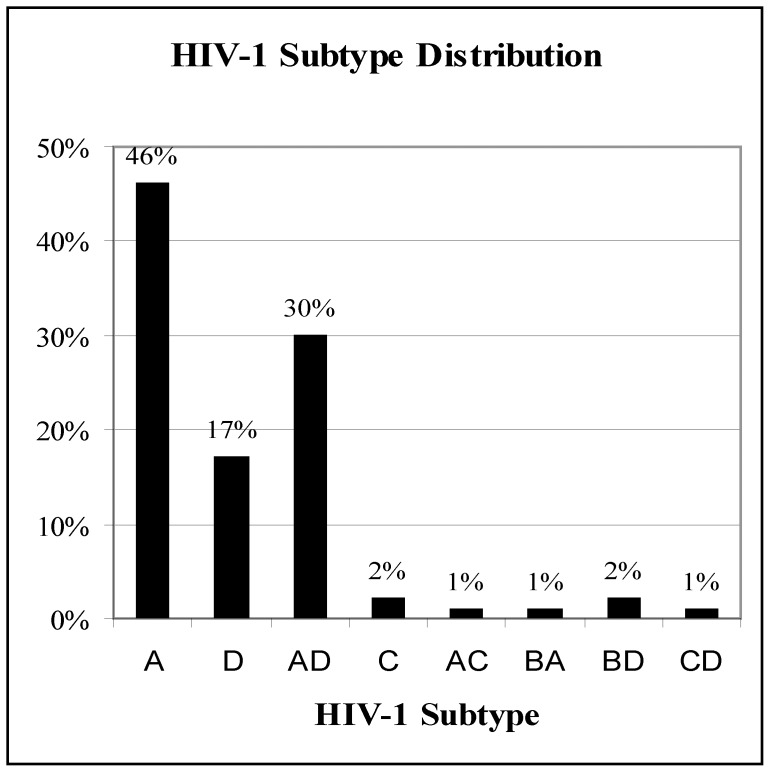
Distribution of HIV-1 subtypes among patients with severe sepsis. Subtype A (n = 81), D (n = 29), AD (n = 53), C (n = 3), AC (n = 2), AB (n = 2), BD (n = 3), CD (n = 2).

**Table 1:** Clinical features of HIV-1-infected patients with subtypes A, D, and AD recombinants.

Variable	A (n = 81)	D (n = 29)	AD (n = 53)
Demographics:			
Age in years; median (IQR)	36 (29–43)	34 (27–40)	33 (24–42)
Number female [n (%)]	39 (48.1)	17 (58.6)	31 (58.5)
HIV Descriptors:			
CD4 cell count in lymphocytes/mm^3^; median (IQR)	60 (12–124)	30 (16–105)	31 (10–81)
Number on HAART [n (%)]	8 (9.9)	3 (10.3)	3 (5.7)
Clinical Variables:			
Admit Karnofsky Performance Score; median (IQR)	40 (30–50)	40 (30–50)	45 (30–50)
Lactate ≥4 mmol/L [n (%)]	30 (37.0)	16 (55.2)	26 (49.1)
Aerobic blood culture positive [n (%)]	6 (7.4)	2 (6.9)	8 (15.1)
*Mycobacterium tuberculosis* blood culture positive [n (%)]	20 (24.7)	10 (34.5)	20 (37.7)

### Phylogenetic Analysis and Subtype Determination

Using ClustalW, the sequences were aligned with those from the HIV sequence database (Los Alamos National Laboratory). The phylogenetic relationship was then analyzed using the neighbor-joining method in the PHYLIP software package. The reliability of the branching orders of the phylogenetic trees constructed were assessed by bootstrap analysis and confirmed by the maximum likelihood method (DNAml) on PHYLIP. The subtypes of the HIV isolates were determined based on their position in the phylogenetic tree in relation to the HIV-1 references. If both *gag* and *env* sequences of a sample were the same subtype, that sample was assigned a single subtype. If, however, the *gag* and *env* sequences of the same sample resulted in different subtypes, this was considered a recombinant. In addition to the above phylogenic analysis, we also utilized the Recombinant Identification Program (RIP) on the HIV sequence database webpage (www.hiv.lanl.gov/content/sequence/HIV/HIVTools.html/) to confirm the subtypes.

**Figure 3:**
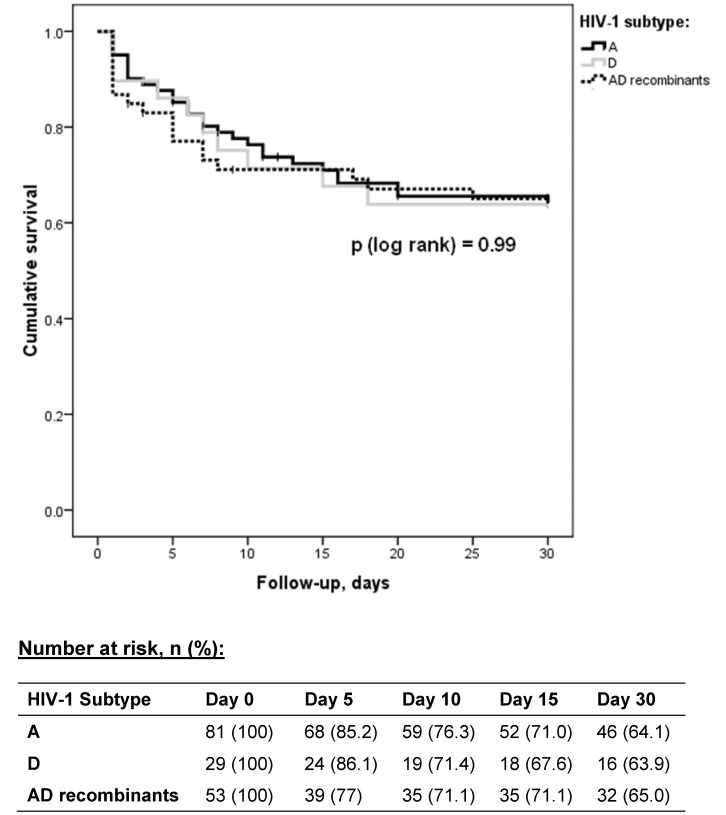
K-M survival curves comparing mortality between HIV-1 subtypes A vs. D vs. AD recombinants.

### Statistical Analysis

Analysis was performed on patients infected with the 3 most common HIV subtypes – subtype A, D and AD. Differences in baseline demographics between the subtypes were compared using a t-test for continuous variables and Chi-square test for categorical variables. Kaplan–Meier estimates of mortality were compared with a log-rank test and length of hospital stay among patients who survived to discharge was compared using a Mann-Whitney test. Cox proportional hazards analysis was used to examine the crude and adjusted relationship between HIV subtype and 30-day mortality. Multivariable analysis identified whether an independent association existed between 30-day mortality and the following independent variables: gender, age, CD4 cell count, HAART use, lactate concentration (< or ≥4 mmol/L), and aerobic and mycobacterial positive blood cultures.

## Results

### HIV-1 Prevalence and Subtype Distribution

Of the 426 patients enrolled in the fluid resuscitation cohort, blood samples were available from 267 patients that were included in this study. Two hundred and twenty eight (85.4%) of the 267 individuals in our study were HIV-1 infected (Fig. 1). HIV-1 subtype determination was successful in 209 (91.7%) of the 228 HIV-1 infected patients. Nineteen samples failed both *env* and *gag* amplification while 34 samples had either the *gag* or the *env* successfully amplified but not both. Figure 2 represents 175 samples where both *env* and *gag* were amplified. The HIV-1 subtypes isolated included A, D, AD, B, C, AC, BA, BD, and CD recombinants, with subtypes A, D and AD recombinants comprising greater than 90% of all subtypes. The most common pattern of AD recombination was subtype A in *env* and subtype D in *gag* (43 out of 53 samples). Of the 34 samples where only one sequence was amplified, 15 had the *env* gene amplified (8 subtype A, 3 subtype B and 4 subtype D) and 19 had the *gag* gene amplified (10 subtype A and 9 subtype D).

**Table 2:** Cox proportional hazards analysis of factors associated with 30-day mortality.

Factor	Hazard Ratio (95% CI)	p-value
Univariate Analysis		
Subtype AD vs. Subtype A	1.03 (0.57–1.87)	0.92
Subtype D vs. Subtype A	1.03 (0.50–2.12)	0.94
Multivariable Analysis		
Subtype AD vs. Subtype A	0.79 (0.42–1.49)	0.47
Subtype D vs. SubtypeA	0.81 (0.38–1.73)	0.59
Female sex	0.85 (0.49–1.47)	0.56
Age	1.00 (0.97–1.03)	0.83
CD4 cell count/10	0.99 (0.95–1.02)	0.39
On HAART	1.16 (0.44–3.05)	0.76
Lactate ≥4 mmol/L	2.02 (1.15–3.52)	0.014
Aerobic blood culture positive*	1.35 (0.50–3.64)	0.55
*Mycobacterium tuberculosis* blood culture positive*	2.19 (1.20–4.02)	0.011

* compared to negative blood cultures.

### Clinical Features of HIV-1 Infected Patients with Subtypes A, D and AD Recombinants

Since the majority (>90%) of the HIV positive patients were infected with subtypes A, D or AD recombinant viruses, analyses were focused on these three subytpes. Baseline characteristics including age, gender, median CD4^+^ T cell count, HAART use, Karnofsky scores, lactate concentration and proportion of positive blood cultures are reported in table 1. The median age and number of females enrolled across the three subtypes were similar. The median CD4^+^ T cell count across all three subtypes was ≤60 lymphocytes/mm^3^ [interquartile range (IQR): 12–124 for subtype A, 16–105 for subtype D, and 10–81 for AD recombinants]. The proportion of patients on HAART was low (9.9% for subtype A, 10.3% for subtype D, and 5.7% for AD recombinants). While the proportion of patients with a lactate concentration ≥4 mmol/L and a positive blood culture for *Mycobacterium tuberculosis* was higher among patients with subtype D and AD recombinants compared to those with subtype A, these differences were not statistically significant.

### Disease Outcome

Disease outcomes recorded in this study included 30-day mortality and length of hospital stay. There was no statistically significant difference in 30-day mortality due to sepsis between patients infected with HIV-1 subtypes A, D and AD recombinants (p [log rank] = 0.99) (Fig. 3). At day 5, it appeared that survival in AD recombinant-infected patients was lower (77%) compared to subtype A- and D-infected patients (85.2% and 86.1%, respectively), however this difference narrowed by day 15 (71% for subtype A, 67.6% for subtype D, and 71.1% for AD recombinants). In addition, among patients who survived to discharge, there was no significant association between median length of hospital stay and HIV-1 subtype (subtype A [6.5 days] vs. subtype D [5 days], p = 0.51; subtype A vs. subtype AD [6 days], p = 0.73).

Results of the Cox proportional analysis to examine the relationship between HIV-1 subtypes, other covariates and 30-day mortality are represented in table 2. Covariates included in the multivariate analysis included age, gender, median CD4 count, receipt of HAART, lactate concentration, and positive blood cultures for aerobic and *Mycobacterium tuberculosis*. None of these factors changed the relationship between HIV-1 subtypes and 30-day mortality (Table 2). However, the multivariate analysis revealed two independent predictors of 30-day mortality – elevated lactate concentration (HR, 2.02; 95% CI, 1.15–3.52; p = 0.014) and blood cultures positive for *Mycobacterium tuberculosis* (HR, 2.19; 95% CI, 1.2–4.02; p = 0.011).

## Discussion

Although the HIV-1 subtype distribution in sub-Saharan Africa has been widely studied, to our knowledge, this is the first study characterizing HIV-1 subtype distribution and its effects in a severely septic cohort. Our findings show a higher proportion of HIV-1 AD recombinant viruses in our cohort of severe sepsis than previously reported non-critically ill cohorts. HIV prevalence in patients with severe sepsis in this cohort was 85% which is similar to earlier studies performed by our group on patients with sepsis in Uganda [21], but much higher than the national HIV prevalence in Uganda (6.5%) [26], and the HIV prevalence among patients presenting to emergency departments in Kampala hospitals (50%) [28]. These data may suggest that infection with HIV-1 increases the possibility for patients to develop severe sepsis.

Data on HIV-1 subtype distribution in clinic patients in the Kampala region of Uganda around the time of our study showed a slight predominance of subtype A over subtype D [12]. Among treatment naïve patients, 49% were subtype A, 28.7% subtype D and 8.1% were AD recombinants. Among the treatment experienced patients, the subtypes A, D and AD recombinant distribution was 41%, 37.9% and 7.4% respectively. While results from our cohort of severely ill patients showed a comparable proportion of patients infected with subtype A virus (46%), a lower proportion of subtype D viruses (17%) and a higher proportion of AD recombinant viruses (30%) existed in our cohort. Without subtype data from a comparison control group of patients without sepsis, our study is limited in being able to definitely conclude whether the enrichment of subtype AD recombinant viruses in our sepsis cohort is significantly greater than the general population of HIV-1 infected patients in Uganda. Notably, the high proportion of subtype AD recombinant viruses among critically ill HIV-1-infected patients has also been reported in a Kenyan cohort in which 6 out of 14 (42.8%) patients with suspected immune reconstitution inflammatory syndrome were infected with HIV-1 subtype AD recombinant viruses [29]. Ultimately, more studies are required to determine if the enrichment of recombinant viruses in critically ill patients is a common phenomenon.

The AD recombinant viruses in these patients could have risen from an initial infection with an AD recombinant virus or from recombination of 2 different viruses that infected the same patient. If the latter is true, infection with multiple HIV-1 viruses might impose a heavier burden on a patient’s immune system, making it more difficult to control other infections leading to sepsis. Recent data from Uganda supporting this theory show that the rate of late stage WHO events was 3 times greater among patients with multiple HIV-1 infections compared to those infected with a single HIV-1 subtype [30].

Previous studies showed that subtype D progressed to AIDS and death faster than subtype A [14], [16], [17]. In addition, patients infected with HIV-1 AD recombinant viruses were noted to progress even faster than subtype D infected patients [14]. Despite the enrichment of AD recombinants in this severe sepsis cohort, we found no significant difference in 30-day mortality between HIV-1 subtypes. In the previous studies, patients were followed prospectively from the time of HIV-1 seroconversion. Our study is limited because patients admitted with severe sepsis only represent a cross-section of the patient’s HIV-1 infection course; thus, the time of HIV seroconversion is unknown. Time to first development of sepsis or frequency of septic episodes may be related to length of HIV seropositivity, rate of CD4 decline, HIV viral load rise and HAART use. Since HIV-1 subtype affects the rate of CD4-cell decline and HIV viral load changes [15], it would therefore affect how early and how often an individual may develop sepsis. Once an individual develops sepsis however, the factors that are most likely to influence mortality due to sepsis are level of immunosuppression and goal directed therapy for sepsis. In this cohort, all the subjects received the same treatment for sepsis [27]. Survival from an acute illness like sepsis seems unlikely to be influenced by HIV-1 subtype. In addition, level of immune suppression represented by CD4 cell count did not significantly affect the relationship between HIV-1 subtype and mortality in this cohort likely due to the overall low level of immune suppression among all sepsis cohort patients (median CD4 count <100 lymphocytes/mm^3^). More studies are required to determine the relationship between the HIV subtype and acute illness.

### Conclusions

This is the first report on HIV-1 subtype distribution in severely septic patients in sub Saharan Africa. We found a high proportion of subtype A and AD recombinant viruses in a cohort of severely septic HIV-1 infected patients from Uganda. The proportion of AD recombinant viruses was higher than previously reported in non-critically ill cohorts. Although it has been shown to affect long-term disease progression, HIV-1 subtype did not affect the outcomes of patients with severe sepsis in our study.
